# Supplementary material for: “Unanswered questions”: Acceptability of a personalised breast cancer screening strategy in lower-risk women by healthcare professionals in the context of the MyPeBS study
Source: PLoS One. 2026 Apr 30;21(4):e0347029. doi: 10.1371/journal.pone.0347029 (PMC13132185; doi:10.1371/journal.pone.0347029)
Supplement: S1 Table — (DOCX) [file pone.0347029.s002.docx]

**Supporting information 2**

**Table 1.** **Views on personalised breast cancer screening**

| **Positive opinion of personalised screening** | |
| --- | --- |
| **Positive view of personalised screening** | *Yeah, I think it’s a great idea and really interesting. Instead of giving everyone the same thing, each person gets their own screening. (P11)* |
| **Improving breast cancer screening** | *(...) for me, the main benefit is improving detection in women we chronically underdiagnose. This includes high-risk women who develop interval cancers, those with dense breasts, or those who have very low sensitivity to the technique. We keep missing cases and only catching them at a more advanced stage (...). (P9)* |
| **Future of breast cancer screening** | *(...) personalisation is making headway. I mean, all healthcare professionals are convinced that it's the only way forward, both in screening and treatment, as well as in diagnosis (...). (P7)* |
| **Positive evaluation among women** | *I think, positively [women's evaluation]. Especially, the high-risk, very high-risk women. Because they see it as ongoing monitoring. (P11)* |
| **Added value to breast cancer screening** | *Especially the genetic test [a novelty]. When you include a genetic test (…), they see it as something extra, on top of what I already do, like mammograms and the medical history I already know (…). They're going to test me for something I don't know about myself, which is this added genetic test. (P7)* |
| **Different care depending on estimated risk** | *(...) I think the fact that it's personalised and there was a previous interview, right? And that they've considered various factors, right? Like the genetic test using saliva, breast density, age, right? I think they appreciate that because it shows we're really looking out for them, you know? It's not the same for everyone just getting a letter at home (...) So I think they feel more cared for. (P11)* |
| **Acceptability of personalization of breast cancer screening in the MyPeBS study** | |
| **Resistance to a change of strategy** | *(...) the success of all our information campaigns to make women aware that they're at risk of breast cancer, in the media, through health information, (...) sends a very strong message. (...) I think most women, whether they have family history or not, are very aware that being a woman means being at risk of developing breast cancer. (P2)* |
| **Factors influencing high acceptance** | *A woman who's never had any family members with breast cancer, never dealt with benign breast issues, and knows her breast density isn't very fibrous, has never heard things like, "you have polycystic or fibrocystic breasts or whatever." For someone like her, who's never really faced breast cancer or benign conditions, acceptance was much easier. (P7)*  *So for me, there is a difference, but not in whether they are younger or older, if they participate in regular screening. It's whether they are already getting screened regularly or not. The ones who had never had a previous mammogram had a very positive acceptance of any schedule you proposed to them. (P8)* |
| **Factors influencing low acceptance** | *The ones with family history are perhaps the most concerned about their potential increased risk of breast cancer (...) women who know they have very dense breasts are also concerned (...). (P2)*  *Yeah, I think it's a cultural thing, right? Yeah, like saying, 'Wow! I might have a higher risk because my great-grandfather, grandfather, and father had cancer (...) Yeah, of course. There's a perception like saying 'yeah, there's a line (...). (P8)* |
