# Supplementary material for: “Unanswered questions”: Acceptability of a personalised breast cancer screening strategy in lower-risk women by healthcare professionals in the context of the MyPeBS study
Source: PLoS One. 2026 Apr 30;21(4):e0347029. doi: 10.1371/journal.pone.0347029 (PMC13132185; doi:10.1371/journal.pone.0347029)
Supplement: S2 Table — (DOCX) [file pone.0347029.s003.docx]

**Supporting information3**

**Table 2. Views on extending screening intervals among women at low risk**

| **Distrust in the proposal to extend screening intervals** | *(...) How do you tell a woman that we're not just trying to save money? That's another thing we get accused of sometimes: You guys don't have enough money, so instead of doing it every year, you do it every 2 years. And now, with even less money, you do it every 4 years. (...) I think a lot of people believe that. (P1)*  *(...) lots of people would say, I have a friend, I know someone (...) who got cancer just 2 months after having a mammogram. So, it didn't make them feel any safer, even doing it every 2 years, because they say cancer is totally unpredictable, totally random. If it happens to you, it happens. (P7)* |
| --- | --- |
| **Prior participation in screening** | *Lots of people felt that every 2 years was already too long, you know? Especially those who were alternating between private and public check-ups. They were like, Now, even longer? Four years? (...) Wow! They're cutting back on my tests. (P8)*  *You can try to tell them: Listen, be well informed about the side effects... If safety is what matters most to you in the end... But here's the thing: you have to respond to the argument, You're giving me less security. Why should that matter to you? Why should it matter to me? Does de-escalation have its benefits? It does have significant positives, but not everyone sees it the same way. (P1)* |
| **Women's sociodemographic characteristics** | *(...) I think the distinction comes down to socioeconomic status (...) and educational level. If you know about medical issues, your own health, and prevention options (...) when this is implemented on a population level, clearly some people will grasp it better and others not as well (...). (P7)*  *(...) And they say: look, we'll do an ultrasound, mammogram, every year, and also whatever else, and for the same package we'll include cytology, and they're delighted. There were ladies who told us they wouldn't come to the program (...) In one insurance company, their package includes mammogram and ultrasound, because it's more comprehensive. (P1)* |
