# Supplementary material for: “Unanswered questions”: Acceptability of a personalised breast cancer screening strategy in lower-risk women by healthcare professionals in the context of the MyPeBS study
Source: PLoS One. 2026 Apr 30;21(4):e0347029. doi: 10.1371/journal.pone.0347029 (PMC13132185; doi:10.1371/journal.pone.0347029)
Supplement: S3 Table — (DOCX) [file pone.0347029.s004.docx]

**Supporting information 4**

**Table 3. Healthcare professionals "doubts” about the implementation of personalized breast cancer**

| **Benefits for low-risk women** | *If we're going to stretch it out to something like four years, we need to be really sure about the percentage of patients who might develop cancer in that time. (…) What’s the sensitivity and specificity? What’s the rate of interval cancers in this group of patients? We need to know these things. To reassure a woman that she’s fine for four years, we need to know the percentage of cancers that would be caught and how many benign lesions would unnecessarily be found. (P9)*  *(...) as long as we have robust, evidence-based results (...) If you tell a woman, 'You're low risk, so we'll do a mammogram every four years,' and then we start finding a lot of interval cancers in this group of women, they're going to completely lose trust in this risk assessment. (P2)* |
| --- | --- |
| **Risk communication** | *Explaining risk is really complicated. When you give the result as a percentage, it might be clear to some people but very confusing to others. (...) You could say, 'You are at low risk,' and they'd ask, 'But... is it zero? Is there no risk at all?' Or you might say, 'You are at low risk because it's 0.2 percent,' and they'd respond, 'Well, that doesn't seem low to me.' Right? Or if it's 0.9 percent and the cut-off is 1 percent, they'd say, 'So I have 0.99 instead of 1. Does that mean every four years?' Even if they understand, they might not agree with the criteria. (P7)* |
| **Women´s participation** | *Equity, right? We already know that the current screening doesn't reach everyone, and making it more complicated will probably result in even less participation or only certain groups taking part. So, adding more complexity means you'll have to accept lower participation levels. (P8)*  *(...) More and more people know about what it means when your DNA is out there in the world, and maybe that's why a lot of people here might refuse. (...) Genetic issues are getting talked about more, more people are getting involved and understanding it, it's becoming more normal to talk about it, and there's more awareness about what I want to happen with it, who should have access, whether they'll just look at this, whether they'll look at more things without my knowing, or whether they'll use it later without my say-so. Collecting samples is tricky (...). (P7)* |
| **Feasibility of implementation** | *You'll need staff who can explain what an SNP* is and understand how to empathise with low or high risks. It won't be easy because if you're actually testing a woman, you need to be able to address her questions—it's your responsibility as a professional. I mean, you can't just tell a woman, 'I'm going to do a genetic test,' and then when she asks, 'What exactly are you looking for?' you say, 'I don't know.' (P11)*  *Nowadays, samples are only collected from those being analysed or because of some illness that´s been detected and needs genetic profiling or something similar. But doing this on a large scale—having labs that can handle this volume of samples, staff who can explain the results, and banks to store these saliva samples... (P7)*  *(...) it's complicated (...) Of course, these two systems [public and private] coexist, there also needs to be a bridge between them (...) because otherwise, we won't be able to maintain this dynamic or active risk calculation over time, if we can't retrieve this information. I think empowering women is great, but from experience, I see that what they report isn't always completely useful or accurate, which can lead to confusion. (P7)*    *(...) they go to their private gynaecologist and they say, Listen, we follow the American recommendations, they know best. And they're very clear that it should be done every year. Public healthcare has to save money here.... That's the message that sinks in. (...) They do what's called dual screening. One year they go to the insurance and one year they come here. (...) We'll do your check-up every year and we'll do the ultrasound and everything. (...) So now it's not just about screening, but about treatment, essential surveillance. (P1)*  *I go to the gynaecologist [publicly-funded system] and say, 'I'm asking for a mammogram because I want to stay on top of things.' Often, the reason for the request is simply 'the patient wants a mammogram. (P10)* |

*** Single nucleotide polymorphism**
