# Supplementary material for: “Unanswered questions”: Acceptability of a personalised breast cancer screening strategy in lower-risk women by healthcare professionals in the context of the MyPeBS study
Source: PLoS One. 2026 Apr 30;21(4):e0347029. doi: 10.1371/journal.pone.0347029 (PMC13132185; doi:10.1371/journal.pone.0347029)
Supplement: Attachment — Submitted filename: Reviewers Comments_Plos One_2.docx [file pone.0347029.s006.docx]

**REF: PLOS ONE [PONE-D-24-54911]**

***“Unanswered questions”: acceptability of a personalised breast cancer screening strategy in low-risk women by healthcare professionals in the context MyPeBS study***

The authors would like to thank the editor and reviewers for their comments, as their contributions have considerably enriched the new version. Suggested modifications have been made to the text, which are highlighted in the manuscript in red. You can find a specific answer to each comment below.

Dr. Paci, for health reasons, I will be away from work for a while. Therefore, I would appreciate it if any messages sent to me could also be forwarded to the other two authors of the article:

**Xavier Castells:** e-mail: [xcastells@psmar.cat](mailto:xcastells@psmar.cat)

**Celmira Laza-Vásquez**: [celmira.laza@udl.cat](mailto:celmira.laza@udl.cat)

Communication 13 June

Please accept my apologies for not responding sooner. As I wrote to the editor in the letter responding to the reviewers on 22 May, I am on medical leave due to health issues and only today did I check the last message sent.

Therefore, I request that all communications be sent to Dr Xavier Castells at [xcastells@psmar.cat](mailto:xcastells@psmar.cat). Dr Castells is the other author of correspondence.

En cuanto a la solicitud:

1. Please provide additional details regarding participant consent. In the ethics statement in the Methods and online submission information, please ensure that you have specified (1) whether consent was informed and (2) what type you obtained (for instance, written or verbal, and if verbal, how it was documented and witnessed). If your study included minors, state whether you obtained consent from parents or guardians. If the need for consent was waived by the ethics committee, please include this information.

Additional information on informed consent was provided both online and in the manuscript. Online, the following was added to the **‘Ethics Statement’** section of the submission: ‘Individual written informed consent was obtained from the participants.’

In the article version, the following was added to section **2.5 Ethical considerations:** Individual written informed consent was obtained from the participants." (page 9, line 186)

I hope that the delay in responding and making the requested modification will not affect the submission and publication process of the article.

Best regards,

**Additional Editor Comments (if provided):**

This is a very interesting and original manuscript. Qualitative research in the context of a randomized trial is still rare. I am convinced of the importance of considering participants' narratives, as the authors have done in previous studies cited in their reference list. This study is original precisely because it considered the experience of the health professionals involved in the MyPEBS study.

1. **I believe that qualitative research incorporated in an intervention study is an added value of the study, but there must be transparent and explicitly stated reference to MYPEBS from the title**.

**RESPONSE:** Thank you for you suggestion. We have modified the title to refer to the MyPeBS project: *“Unanswered questions”: acceptability of a personalised breast cancer screening strategy in low-risk women by healthcare professionals in the context MyPeBS study.* (page 1)

**So, if the manuscript is revised in this direction, in my opinion, there is no such problem. The separation of qualitative researchers from research participants is sufficient, in my opinion, to ensure autonomy and allow for work that might otherwise be difficult to accomplish.**

**2. I agree with the reviewer that, given the context, the personal identity of respondents working in a single hospital should be protected more carefully. Table 1 should be eliminated, and some information about participants, in aggregate, should be presented as text.**

**RESPONSE:** Thank you very much for your suggestion. We appreciate the attention that you and reviewer 2 have given to this issue. We agree to remove Table 1 from the study participant characteristics and include the following description of participant characteristics in the text (page 7 line 140):

*“Fourteen healthcare professionals participating in the MyPeBS project in Spain were invited in person and/or by email, and three did not respond. The sample included 11 professionals with an average of 15.5 years of experience in the breast cancer screening programme (range 2 to 28 years), with different professions and roles within the project: three doctors specialising in radiology and one in gynaecology and obstetrics; one doctor specialising in public health and epidemiology; four senior diagnostic imaging technicians; and two research technicians responsible for recruiting women for the study. 73.7% of the subjects were women and the average age was 40.7 years (range 25 to 65 years)”.*

**3. It is also necessary, in agreement with the reviewer to specify what is meant by reaching theoretical saturation. From the way it is presented it may appear that there are a limited number of professionals to interview.**

**RESPONSE**: Thank you for you suggestion. It is evident that both you and reviewer 2 are correct in their assertions. When a predetermined number of participants is established, the criterion of information saturation does not dictate the size or configuration of the sample. However, it should be noted that, despite the limited number of participants in the study and the heterogeneity of their profiles, the information became saturated. The analysis, in conjunction with the collection of information, demonstrated theoretical saturation of all topics commencing with participant number 8.

Therefore, the statement *“The sample size was determined using the criterion of theoretical saturation (17)*” was removed.

However, we have made the following clarification in the text (page 7 line 150): … *“and from informant number 8 onwards, no new information was obtained that would permit further expansion or deepening of the results. This finding suggests that theoretical saturation of information was achieved”.*

**4. I consider the text to be too long and ask the authors to limit themselves to presenting their interesting results, cutting out all the sections about the prospects for future work. These, in my opinion, are conventional and in any case could be better developed in a later manuscript that takes into account the whole of the qualitative research work done on the MyPEBS study, rather than being presented as a consequence of the health practitioners' evaluations alone.**

Response: Thank you for you suggestion. We agree with the suggestion, so we have removed Theme 4. Recommendations for the implementation of personalised breast cancer screening in Spain.

We have also made the following modifications:

- Removed Table 5 “Table 5. Recommendations for the implementation of personalised breast cancer screening in Spain”.
- The objective in the abstract and the introduction was modified. It was also included that the context of the study was the MyPeBS project (page 2 line 29 and page 6 line 114): *“We explored healthcare professionals involved in the MyPeBS study views on the acceptability of a personalised strategy for breast cancer screening in women deemed to be at low-risk”.*
- In the abstract removed the results on the recommendations of health professionals: *“To promote acceptability, they recommended "staggering" implementation, training of healthcare professionals, and development of socialization strategies.”*

In the discussion, some sections on the discussion of the results of topic 4 of the results were removed:

*Our results are consistent with previous approaches and, given the above-mentioned difficulties, proposes innovative solutions such as the creation of "information exchange networks" to facilitate the regular training of primary care professionals. This is a novel and undocumented recommendation in the scientific literature, where only online courses, websites and seminar-style conferences are proposed(Ayoub et al., 2023; Lapointe et al., 2022).*

*An issue not highlighted by the healthcare professionals participating in the study, but widely discussed in the literature, is the integration and involvement of nurses in the implementation(French et al., 2022; Lapointe et al., 2023b; Laza-Vásquez, Hernández-Leal, et al., 2022). This is a key consideration because of nurses’ skills and their contributions to the development of current screening programs(Bakhai et al., 2024). In addition, their involvement would help to "spread" the workload in primary care teams(Ayoub et al., 2023; Laza-Vásquez, Codern-Bové, et al., 2022; G. Taylor et al., 2022).*

*For the purpose of informing and educating women, a relevant aspect of this study is the participants' proposal to amplify "women's voices" to help the socialisation of personalisation through women-to-women dialogue. This approach is based on the "experiential knowledge" of lay women and the scientific knowledge of experts. This strategy could be valid for the socialisation of personalisation given its known benefits in raising public awareness of breast cancer through patient associations and "expert patients"(Foucaut et al., 2023; Galvin et al., 2022; McDonald et al., 2023; Rivest et al., 2020) and for survivors of the disease as a valuable source of information and support(Fong et al., 2017; Harmon et al., 2021) and by fostering a "sense of community" among women(Buki et al., 2023).*

*Finally, and in line with other studies, participants supported a proposal for a "staggered" implementation. Several elements support this suggestion: women already participating in screening programmes are "familiar" with this model(L. C. Taylor, Law, et al., 2023) and breaking the "promise" of biennial screening could create mistrust(Woof et al., 2021) and potentially provoke feelings of "injustice" among women(LA Dunlop et al., 2024). Conversely, less intensive screening would be more acceptable to younger women, as they are less aware of current screening intervals(Woof et al., 2021) and consider the disease to affect women older than 50 years (Hindmarch et al., 2023) and mammography to be a "cumbersome" examination(L. C. Taylor, Law, et al., 2023).*

**And others were modified:**

- Page 18 line 404: “*Therefore, to promote acceptability among women deemed to be at low risk, several recommendations made by other researchers should be taken into account:…”*
- Page 19 line 438: *“In order to inform, educate and socialise women about personalised screening strategies, community initiatives such as patient associations and the “expert patient” strategy could be beneficial, as they have proven to be valuable sources of information and support and promote a ‘sense of community’ among women”*

**5. Reviewer 2 raises a central point because here the central issue is not his perception of risk (population risk) but the individual one, relative to the specific individual. On this basis, " accepting that some women, both women at higher risk and lower risk, will accept greater risk of harms for greater assurance of safety," and what is the purpose of risk stratification, what kind of information is needed to illustrate in the real and individual context the knowledge of the natural history of the tumor that allows the attribution of risk should be investigated. In the end, as the reviewer suggested, there is need to be explicit, also in a quantitative manner, with the existing evidence that conditions the individual decisions.**

**RESPONSE:** We appreciate the suggestion, which, as researchers, makes us reflect on the urgency of future studies that use mixed approaches and/or methodological triangulation processes to obtain more comprehensive and in-depth results. However, a qualitative approach was proposed for this study precisely because it was conducted in the context of the MyPeBS randomized trial, in which a quantitative analysis is being carried out.

Therefore, we emphasise presenting the qualitative findings in a comprehensive and in-depth manner that reflects their richness and the different points of view and perspectives from the diverse experiences and roles of the participants. Perhaps, although the narratives may seem lengthy, it is precisely these broad, in-depth narratives, in which all points of view are respected, that achieve the objective of qualitative research**.**

**Reviewer 1 comments**

The authors explored the opinions and thoughts about implementation of personalized breast cancer screening in Spain through interviews with a variety of health care professionals.

1. **In the introduction, the authors state, “Risk-stratified breast cancer screening is a promising strategy aiming to optimize the current model [of breast cancer screening] by enhancing the balance between the benefits and harms of early detection.”**

**Well, yes, but what exactly is meant by “enhancing” the balance of benefits and harms? What is missing in the introduction is a discussion of the inherent conflict in current models of risk stratification between benefit to risk balance on an individual basis and a population basis, including accepting that some women, both women at higher risk and lower risk, will accept greater risk of harms for greater assurance of safety.**

**RESPONSE:** Thank you very much for the suggestion. We have included information to clarify the statement.

*… by increasing sensitivity in women at higher risk of breast cancer and specificity in women at lower risk, with an overall balance no worse than current screening. (Page 4 line 56)*

*… while decreasing screening frequency and harms such as overdiagnosis and false positive results. (Page 4 line 60)*

2. As a prelude to discussing the responses from health professionals, the authors should briefly describe the trade-offs between benefit and harm in some models of risk stratification, the current state of evidence for the key risk factors that would be applied to risk stratification, and most especially, the absence of data that the major risk factors are associated with the breast cancer sojourn time. Just because a woman is less likely to develop breast cancer during a 1, 2, 4 (what happened to 3?) year interval, it does not mean that her breast cancer will also grow more slowly, so that the screening interval assigned to various risk profiles will always be shorter than the mean sojourn time. This would be first step towards being able to provide reassurance. It also should be noted that biennial screening already is not risk-stratified for pre-menopausal women, since the combination of density and somewhat faster tumor growth increases the risk of being diagnosed with an advanced breast cancer. **What is missing in this introduction is the importance of setting the stage for the discussion of the professional’s opinions. What also is missing is whether the principle of risk stratification in this context is intended to achieve the principle of “equal treatment for equal risk,” i.e., that the goal of risk stratification would be to efficiently provide all women with an equal opportunity to have their breast cancer detected at an early stage. If the model favors high risk women on this principle over lower risk women (who, actually, have comparatively high lifetime risk compared with other chronic conditions), then it should not be surprising that many women would have less interest.**

**RESPONSE:** We appreciate the suggestions made to improve the introduction to the article, with which we agree. Upon rereading, we also consider it necessary to include information on the opinions of health professionals regarding the personalised breast cancer screening model and, in particular, on their experiences with women who are considered to be at low risk. We have therefore included the following information (page 5 line 96):

*“There is limited evidence on the opinions of healthcare professionals involved in personalised screening projects regarding the acceptability of this strategy. In general, opinions are positive, considering it a logical step in breast screening. They believe that this strategy will particularly benefit high-risk women by increasing the frequency of screening, reducing mortality from the disease and increasing the survival of women diagnosed. For low-risk women, they report a reduction in overdiagnosis and false positives. These advantages will increase the efficiency and effectiveness of the use of healthcare system resources. The main disadvantages highlighted are the potential resistance of low-risk women to the recommendation to reduce the intensity of mammographic screening.*

*Only the BC-Predic project in the United Kingdom has thoroughly investigated the views of those involved on reducing screening intensity. Health professionals consider this logical, but they are cautious in recommending it due to doubts about the accuracy and stability of risk estimates and the increase in interval cancers, as well as a misperception among women that low risk is synonymous with no risk”.*

Furthermore, we would like to point out that this article is not the place for debate, which we consider very necessary, on the merits, benefits and disadvantages of personalised breast cancer screening models, or on the evidence regarding the factors used by different models to calculate and stratify risk. We are convinced that future publications of the results of the MyPeBS project will allow these aspects to be evaluated and discussed.

**3. I would then characterize the response from the health professionals in a more quantitative manner, especially to the extent that their opinions are consistent with the prevailing evidence. The responses are interesting, and I would retain the most illuminative opinion statements, but the narrative is rather long as submitted.**

**This is a novel study, but it should be grounded in the current evidence (this would benefit the reader), the variability in the concept of risk-stratified screening and expected outcomes on a population and individual basis—in most models, there are winners and losers—the higher costs of risk of errors in establishing and regularly updating risk, and then how this health professionals understand and feel about this concept.**

**RESPONSE:** We appreciate the suggestions, which have already been addressed in the suggestions made by the editor (see response to the editor's comment number 5).

**Reviewer 2 comments**

**Thank you for the interesting reading of your paper.**

The paper poses an important research question to succeed the implementation of a new perspective of population-based screening which leave the concept of “a one size fits all program” to a complex concept of risk-stratified programs adjusted to fit the individual risk. Health professionals are important stakeholders in risk information and may have to answer questions while working in related areas.

**1. This study planned to interview 14 healthcare professionals participating the MyPeBS project in Spain of whom 11 participated. Four in a focus group interview and 7 in individual interviews. Three did not participate. The healthcare professionals are not representative of the health care community because they work in breast cancer screening and are involved in MyPeBS. It would suit the paper to clearly state this in the discussion and as a limitation.**

**RESPONSE:** Thank you for your feedback and suggestion.

We agree that the participating professionals are not representative of the healthcare community, but we do not consider this to be a limitation of the study. The aim of the study was to gain an insight into the opinions of professionals involved in a MyPeBS study and thus reflect what a situation might be like where a personalised breast cancer programme is implemented. This is an added value, as the opinions were obtained in a real context of personalisation rather than a hypothetical one, as we highlight in the section on the strengths and limitations of the results.

We would also like to highlight that a characteristic of qualitative research is the configuration of relatively homogeneous samples (in this case, homogenisation is participation in the MyPebs study) that allow the construction of ‘patterns’ in the emerging discourses.

**2. Do you use pseudonymization to present your participant?
I am ethically worried about the 11 participants being identified by colleagues, patients and people in Hospital del Mar? Breast cancer Screening is a small area of healthcare and the detailed information you provide make me picture all of them. Please, specify whether pseudonymization was used. It gets worse because all quotes are referred to a given participant number. Please, omit that from result tables. That information is not needed by the reader.**

**RESPONSE:** Thank you for you appreciation. No pseudonyms were used to identify study participants; instead, they were assigned a numerical code known only to the researchers. Identification using a numerical code was approved by the Ethics Committee and accepted by the participants (this issue was clarified during the informed consent process).

We understand your concern about the difficulty of maintaining the confidentiality of participant information in such a small setting. Therefore, we have removed Table 1, as recommended by the editor, from the participant characteristics and inserted a paragraph about them (see response to the editor's comment number 2).

**3. The result section is elaborate and present at detailed view into your data. However, the section is too long, and the reader is left without an overview of the important findings. I suggest the tables to be put in a supplement. Keep Table 1 an a few important quotes.**

**RESPONSE:** Thank you for your suggestion. The tables of codes and verbatim quotes for each topic were removed from the text of the article and included as supplements. Table 1, at the suggestion of reviewer 1 and the editor, was removed from the text of the article and a description of the characteristics of the participants was provided.

We have followed your suggestion to send the tables of verbatim quotes as supplements.

**4. The discussion could benefit from knowledge about the general practitioner’s role in screening communication. They have an important role in many people's life because they seek help in their local health environment.**

Response: Thank you for your suggestion.

One of the determining factors in the future implementation of personalised breast cancer screening as a population-based programme will be the role played by primary care professionals in raising awareness of the screening model and inviting women to participate. However, in the European context, and particularly in Spain, primary care professionals are generally not very involved in population-based early detection programmes, as invitations and socialisation are carried out directly by the population-based screening programmes.

We reiterate that the involvement and participation of primary care and its professionals will be key to the implementation of personalised population-based programmes. In fact, this issue is highlighted by the participants in the study's findings. We emphasised this point in the discussion (page 18 line 419):

Another essential and pressing issue raised in the study is the training of primary care *professionals is the need to train primary care professionals, given the decisive role they will play in implementing a personalised screening model*. Two large studies reported that these professionals lack sufficient knowledge and need further training. Dunlop et al emphasised that training them to carry out a change in practice would be a challenge that involves taking into account their diverse activities and roles, and time constraints.

**5. Finally your reference list is impressing. By thorough reading I find most papers published by the researchers involved in MyPeBS collaborating across institutions. Perhaps, it is dose not pose a problem however, the references are used as examples of agreement with other researchers in the discussion and that is not convincing reading. Please, specify when co-workers are sited and use more evidence from other research groups if possible. Otherwise cut down on citations.**

Response: Thank you for you appreciation. We have reviewed and reduced the list of references (12 references). We reviewed the references and removed several of them. Regarding the authors' publication references, we would like to emphasise that all three authors participated in the only two studies conducted in Spain on personalisation of screening (DECIDO and MyPeBS). We therefore consider it important to cite them in the article.

Following this suggestion, we have removed several citations from articles published by the authors when there were citations from other studies that supported the statement in the text.

**6. The paper is well written and structured and easy to understand. Minor language revisions are needed.**

Response: Thank you for you appreciation. The text, after modifications made taking into account the evaluators' suggestions, was reviewed in its entirety.

**7. Detailed feedback**

**- Line 69, page 4 Personalization increase complexity. Please, specify complexity of what?**

Response: Thank you for you appreciation. We would like to clarify the following (page 4- line 69)

*“However, personalisation increases complexity due to the socialisation of the message and its communication to women, which makes the role of healthcare professionals essential in explaining complex concepts such as relative and absolute risk reduction, overdiagnosis and false positive results. Furthermore, it is expected that screening intensity will increase in high-risk women and decrease in low-risk women. Furthermore, complexity increases in the future implementation of this strategy as a population programme”…*

**- Line 72, page 4 “the potentially lower of extending” ? Do you mean acceptance. Please, correct the sentence.**

**RESPONSE:** Thank you very much for identifying this error. It has been corrected: … the potentially lower *acceptance* of extending screening… (page 5 line 79)

- **Line 75, page 4 Brackets contain reasons for acceptance. Should habits be included?**

**RESPONSE:** Thank you for you appreciation. Sin embargo, the current literature does not describe lifestyle habits that influence the acceptability of the screening model or a personalised model.

- **Line 108, page 6 An actual status of the recruitment as of today would be relevant. The paper states that 53,142 women aged 40 to 70 y were recruited by mid-2023. Visiting the webpage as of March 28th, it is stated that 53,143 women are recruited. Please clarify whether that means nobody has been recruited since mid-2023? Or the study has stopped recruiting.**

**RESPONSE:** Thank you for you appreciation. The article clarifies this point (page 7 line 134):

*“Recruitment was completed”*

- **Line 132, page 9. “The sample size was determined using the criterion of the theoretical saturation.” How did you do that when the 14 healthcare professionals were selected up front?**

**RESPONSE:** We appreciate the suggestions, which have already been addressed in the suggestions made by the editor (see response to the editor's comment number 3).

- **Line 139, page 9 The interview script? Do you mean a structured interview-guide? The answers were open for the participants?**

Response: Thank you for you appreciation. We would like to clarify that a semi-structured interview was used as the data collection technique (page 8 line 156):

*“Participants were interviewed, using a semi-structured interview guide…”*

- **Line 225, page 16 The heading is mistrust but distrust is used in Table 3, second question.

  Table 3 In the text page 16, you make headings for three themes, but the table contains four views.**

**RESPONSE:** Thank you for you appreciation. We have removed the first code from Table 3 (now Table 2) “Estimation of low **risk”**

**- Section 3.4.1 This section could be shorter because it reflects the opinion of healthcare professionals attitude towards risk communication to citizens. Please specify, that they are laypeople in this professional field. Professional communicator can write a better and more effective communication strategy for implementation of risk-stratified breast cancer screening. Some details are not important.**

**RESPONSE**: Thank you for you appreciation. As explained in the response to the editor, topic 3 has been removed and the most relevant aspects of the health professionals' recommendations are presented in the article.

**- Line 544-48, page 42. Can risk-communication be specified whether it’s SNP’s or high penetrating genes we communicate about. Studies are often divided into one or the other and the findings are not identical.**

**RESPUESTA:** Thank you for you appreciation. Is it possible to estimate breast cancer risk using single nucleotide polymorphisms (SNPs)? By themselves and individually, SNPs cannot estimate the probability of developing breast cancer, as high-penetrance genes can. However, it is possible to estimate the probability of breast cancer by calculating a polygenic risk score from the combined effect of single nucleotide polymorphisms (SNPs) related to the disease (Evans et al 2016). Currently, more than 313 SNPs related to the disease have been discovered. Information on SNPs, combined with factors such as family history of breast and/or ovarian cancer, personal history of benign breast lesions, hormonal information, and breast density, allows for an accurate estimation of breast cancer risk (van Veen et al., 2018).

SNPs also explain a large proportion of inherited genetic risk in women developing sporadic breast cancer and having no familial history of the disease. Therefore, at population level, SNPs are likely to be more informative for risk stratification than screening for moderate- and high-risk gene variants in a gene panel; and recent studies have considered the value of including an SNP-based PRS in risk prediction algorithms, showing promising results. (Evans eta al., 2022).

- van Veen EM, Brentnall AR, Byers H, et al. Use of single-nucleotide polymorphisms and mammographic density plus classic risk factors for breast cancer risk prediction. JAMA Oncol 2018; 4(4): 476–482.
- Evans et al. Improvement in risk prediction, early detection and prevention of breast cancer in the NHS Breast Screening Programme and family history clinics: a dual cohort study. Southampton: NIHR Journals Library, 2016, pp. 4–11.
- Evans et al. Breast cancer risk stratification in women of screening age: Incremental effects of adding mammographic density, polygenic risk, and a gene panel. Genet Med. 2022 Jul;24(7):1485-1494.
