# Supplementary material for: “Unanswered questions”: Acceptability of a personalised breast cancer screening strategy in lower-risk women by healthcare professionals in the context of the MyPeBS study
Source: PLoS One. 2026 Apr 30;21(4):e0347029. doi: 10.1371/journal.pone.0347029 (PMC13132185; doi:10.1371/journal.pone.0347029)
Supplement: Attachment — Submitted filename: Reviewers Comments_Main_4_Plos One_2025.docx [file pone.0347029.s007.docx]

**REF: PLOS ONE [PONE-D-24-54911]**

***“Unanswered questions”: acceptability of a personalised breast cancer screening strategy in low-risk women by healthcare professionals in the context MyPeBS study***

The authors would like to thank the editor and reviewers for their comments, as their contributions have greatly enriched the new version. The suggested changes have been incorporated into the text and are highlighted in red in the manuscript. Below is the response to the editor's and reviewer's suggestions.

**Additional Editor Comments:**

**Thank you for the revision and changes in the title and manuscript. I think the manuscript is now more readable, and you avoid the problem of the conflict of interest and the issues about privacy.**

**Still, there are problems, as the reviewer underlines in its second revision, you should try to better address. These issues are at the core of the message of your paper and condition the meaning of the paper for the journal's audience.
So, I strongly suggest not avoiding the issue and trying to argue the comment of the reviewer.**

**There is no contrast between a population-based approach versus an individual approach. In this kind of evaluation, we always apply individual estimates based on estimates from population studies.**

**The issue at the core of the discussion, in my opinion, and I guess in agreement with the reviewer, is that this qualitative assessment occurs in the context of an RCT, MyPeBs, which is aimed at overcoming the limited knowledge we have about personalized risk.**

**I did not read any consideration from the professionals about the level of uncertainty being the reason why a woman is recruited in a trial like this.
This is particularly true on the inter-screening interval. In the personalized assessment, we can define a women as at low risk given for example a biomarker negativity.**

**Does that mean that lower risk means slower tumor growth?. More data are needed; this study will contribute to this, we hope. However, I should ask to be very clear about this point. Whereas sensitivity is important and personalised screening will primarily contribute to better detection, we must be very careful about communication of what we do not know both in the safety of screening in lower risk women, both about side effects, which we do not really know in the new context of risk based screening (false positive and, most, overdiagnosis)**

**In conclusion, I invite you to a revision considering these and the reviewer's suggestions both in the text and in the abstract.**

**Reviewer #1**

**I'm disappointed that the authors have not addressed my comments, specifically that they overlook the inherent problem that personalized screening is commonly conceptualized as improving detection of breast cancer in higher risk women, and reducing recalls in lower risk women. As they say very early in the introduction, "by increasing sensitivity in women at higher risk of breast cancer and specificity in women at lower risk, with an overall balance no worse than current screening." Is this progress? Not in my opinion. We need to do a much better job at detecting breast cancer early in all women.**

**And we need to do this more efficiently, which means also reducing avoidable recalls. What is seriously flawed in this study is that health care professionals were interviewed about screening concepts for which they are not well informed, but demonstrated, I think, an impressive degree of caution about the potential costs of personalized screening to lower risk women. The authors should also have shown greater awareness of the limitations of our current risk models, and here is the main point, the very weak data linking risk and sojourn time.**

**The authors seem to believe that women's concerns about being diagnosed with a worse prognosis cancer when the screening interval is lengthened can be addressed with better education about the advantages of avoiding overdiagnosis and false positives, setting aside that we do not have any estimates of breast cancer overdiagnosis that we can cite with confidence, let alone overdiagnosis associated with the common risk factors.**

**In my review, I asked simply that the authors note that we do not have any data that demonstrates that the primary goal of screening, which is to detect the killing cancers early, can be improved for all women with a personalized model that screens higher risk women more frequently and lower risk less frequently. For some reason, the current thinking is that more screening for some women has to be balanced with less screening for other women. There is no such requirement, nor do we have the knowledge to extend screening to 3 and 4 year intervals in lower risk women without resulting in increasing their risk of being diagnosed with an advanced breast cancer. It is a worthy goal to determine how to screen some women less frequently safely. But all women deserve screening that delivers improved sensitivity and specificity. Until we can do that, the priority has to be sensitivity.**

**Response:** We appreciate the suggestion from the reviewer and editor.

We agree that there is insufficient evidence to support the benefits of personalised screening compared to the current screening model. As the reviewer points out, increased screening for some women is not necessarily offset by reduced screening for low-risk women, and we cannot guarantee that there is no risk of detecting cancers with a poorer prognosis if the screening interval is increased to 3 or 4 years.

Precisely, the research carried out by the MyPeBS study aims to provide evidence on this issue. In the meantime, we start from a position of uncertainty, as the reviewer comments. However, we do start from the hypothesis that personalised screening will lead to an improvement, and this is what ethically justifies the MyPeBS study.

We would also like to point out that the qualitative study aimed to describe the opinions and acceptability of healthcare professionals regarding the reduction of screening for women who are at low risk of breast cancer. Like researchers from the BC-Predict Project in the United Kingdom, who, as in our case, have investigated this issue with healthcare professionals and women in a real context of personalised screening:

- *Taylor G, McWilliams L, Woof VG, Evans DG, French DP. What are the views of three key stakeholder groups on extending the breast screening interval for low‐risk women? A secondary qualitative analysis. Health Expect. 2022;25(6):3287. 10.1111/HEX.13637*
- *McWilliams L, Woof VG, Donnelly LS, Howell A, Evans DG, French DP. Extending screening intervals for women at low risk of breast cancer: do they find it acceptable? 2021;21(1):637. 10.1186/s12885-021-08347-w*
- *Woof VG, McWilliams L, Donnelly LS, Howell A, Evans DG, Maxwell AJ, et al. Introducing a low-risk breast screening pathway into the NHS Breast Screening Programme: Views from healthcare professionals who are delivering risk-stratified screening. Women’s Health. 2021;17:17455065211009746. doi: 10.1177/17455065211009746*

We are also aware that the qualitative study was conducted in a context of uncertainty regarding the evidence on the benefits of a personalised breast cancer screening strategy and that, as it is considered a promising strategy, two randomised studies are currently being conducted to test the superiority of the current screening method. Therefore, we agree with the reviewer's recommendation on the need to be explicit in clarifying this level of uncertainty in the current evidence, and we have made the following changes to the manuscript to provide greater clarity:

**Abstrac (Page 2 line 27):**

“*Based on the relevant differences in breast cancer risk, strategies for personalizing population screening have been proposed. The present study was conducted in the context of the MyPEBS study, which seeks to improve the evidence on the benefits and risks of implementing personalized screening. Specifically, it evaluates the acceptability among professionals of the strategy of increasing the screening interval in low-risk women”.*

**Introduction (Page 4 line 56):**

“*The risk of developing breast cancer over a woman's lifetime depends on characteristics such as age, breast density, family history of breast cancer, history of benign lesions, or genetic predisposition, among other factors. The difference in risk can be substantial between two women based on these characteristics. However, population-based breast cancer screening programmes are based solely on age. It is in this context that it has been proposed to evaluate the effectiveness of screening based on a woman's individual risk [1].*

*There is still no evidence to suggest that personalized breast cancer screening is no worse than or potentially better than current screening in reducing breast cancer mortality [2]. Specifically,* there is insufficient evidence to demonstrate that the primary goal of screening, which is to detect the killing cancers early, can be improved for all women with a personalized model that screens higher risk women more frequently and lower risk less frequently. *However, it is suggested that this strategy could increase the benefits and reduce the harms of current screening; therefore, two prospective multicenter randomized controlled trials are being developed internationally to generate evidence on the effectiveness of personalized screening in reducing breast cancer-specific mortality compared to current screening (Wisdom in the US and MyPeBS in Europe and Israel) [3].*

**Page 5 line 82:**

*“One of the limitations of implementing personalised screening is ensuring similar sensitivity to the current level in low-risk women who are offered longer intervals between screenings. It is expected that additional examinations will be reduced if the screening interval is increased, but this should be done while ensuring equal sensitivity in this group of women”.*

For our part, in the discussion section, we highlight this fact, which both the study participants and we as researchers consider to be of great importance:

**Page 16 line 365:**

*“This study was conducted in the context of the MyPeBS study, which aims to improve the evidence on the effectiveness of a personalised screening strategy based on individual risk. Therefore, there is no robust evidence to show that personalised screening is better than the current strategy based solely on age. However, the hypothesis is that personalised screening will increase sensitivity in women at higher risk without compromising sensitivity in women at lower risk, for whom the screening interval is increased. In fact, the aim of this study is to analyse the opinion of healthcare professionals, assuming that this hypothesis is met, but with the uncertainty that it has not been confirmed”.*
