# Supplementary material for: “Unanswered questions”: Acceptability of a personalised breast cancer screening strategy in lower-risk women by healthcare professionals in the context of the MyPeBS study
Source: PLoS One. 2026 Apr 30;21(4):e0347029. doi: 10.1371/journal.pone.0347029 (PMC13132185; doi:10.1371/journal.pone.0347029)
Supplement: Attachment — Submitted filename: _RESPONSE to Reviewers Comments_Main_5_Plos One.docx [file pone.0347029.s008.docx]

**REF: PLOS ONE [PONE-D-24-54911]**

***“Unanswered questions”: acceptability of a personalised breast cancer screening strategy in lower risk women by healthcare professionals in the context of the MyPeBS study***

**Additional Editor Comments:**

I received the comment to the revision and the review is favorable, but still there are suggestions which are interesting. I ask the authors to consider the new comments and to consider them it in the revised text or writing a comment.

Reviewer #1: Thanks to the authors for their response to the reviewer's comments. [Note to the authors—lower-risk vs. higher risk is more appropriate in this context, and does not diminish the distinction between lower risk and higher risk women, or the purpose of your study. Even what you are calling a low-risk women carries a non-trivial 10-year and lifetime risk. We know very little about what explains never being diagnosed with breast cancer over the course of a lifetime, or how risk evolves in some women, and does not in others].

We appreciate the authors providing what they believe is corroborating information, but it seems the supporting references mainly are from like-minded investigators who believe that the pathway to improving the balance of benefits and harms for all women, is more sensitive screening (with whatever that would require) in higher risk women, where we also are more accepting of harms in the context of that risk, and less sensitive screening (here I’m referring to program sensitivity) in lower-risk women. Indeed, the citations include many of the same authors, so it is not surprising that the themes are similar, and key issues consistently are not addressed.

• Taylor, et al. stresses that the harms of screening low-risk women, who could experience greater harms from overdiagnosis. This is a common theme that low-risk women tend to be diagnosed with low-risk cancers. No evidence is offered for this belief, nor is the issue addressed of how low-risk, and thus lower incidence, can result in greater rates of overdiagnosis.

• McWilliams, et al. This paper offers a similar approach as the others….the pathway to less frequent screening among low-risk (sic) women is providing reassurance of precision in risk estimation and safety, and endorsement from their health care providers. As with the others, the ability to estimate risk is sufficient; correlation with risk and the aggressivity of tumors is assumed.

• Woof, et al. opens with an unproven assertion that low-risk women are more likely to be diagnosed with low-grade and in situ tumors, and that for every breast cancer death prevented, 3 cases are overdiagnosed (the UK report, and a largely meaningless statistic). They state, “it is argued by some that the benefits of screening outweigh the harms.” Really? Argued by some? And, this side of the argument is balanced by a letter to the editor by Donzelli following the UK Independent Review stressing that breast cancer screening does not reduce all-cause mortality. Who knew?
In these three papers, the issue of numeracy (what does it mean to have a 2% risk over 10 years?...it means 1 in 50 women will be diagnosed with breast cancer) and how well do clinicians understand the issues of sojourn time, tumor characteristics, and absolute risk (not RR) and they lack parallel evidence to answer these question for the lower risk group vs. the higher risk group.

On page 2, line 27, the authors highlight revised text that states that the present study was conducted in the context of the MyPEBS study, “which seeks to improve the evidence on the benefits and risks of implementing personalized screening. Specifically, it evaluates the acceptability among professionals of the strategy of increasing the screening interval in low-risk women”.

Of course this is important, but we know that the average health professional is as uninformed about these issues as their patients. The authors seem to be putting the cart before the horse. The evidence that lower-risk women can be screened safely at wider intervals requires screening outcome data before there can be a public health campaign to influence clinicians and the target population. It is quite ok to plan in advance for that campaign, in fact it is the responsible thing to do, what the planning should consider all outcomes, not only for the outcomes they hope to observe.

**Response:** We appreciate the suggestion from the reviewer and editor. We have reviewed the reviewer's proposed arguments in detail. And we agree with them. We have modified the text throughout the manuscript (marked in red) to accommodate this idea. Given that MyPeBS proposes increasing the screening interval in lower risk women, it is essential to quantitatively evaluate the evidence on the safety of increasing this screening interval, and the type of tumors that might be diagnosed in this risk group.

**New text**: Please, see proposed text (marked in red) throughout the manuscript.

**New text** (Discussion, page 21): Emerging evidence supports the feasibility of risk-based breast cancer screening. The WISDOM Study has demonstrated that personalized screening can effectively stratify women according to their risk while remaining safe and acceptable to participants (38). These findings provide essential support for the transition from age-based to risk-stratified screening strategies, although further refinement of risk prediction models and risk-reducing recommendations as well as effective risk communication to patients and health care professionals are still needed for large-scale implementation. Importantly, reducing the frequency of screening in women at lower risk needs to be monitored to avoid losing sensitivity in the detection of early stage breast cancers.

**Page 5, line 82: “…**It is expected that additional examinations will be reduced if the screening interval is increased, but this should be done while ensuring equal sensitivity in this group of women”. Good. But, suggest that the authors say “equal sensitivity in the detection of early-stage breast cancers by screening in this group of women.” Sensitivity may be minimally diminished, but tumor characteristics could be worse. In other words, in addition to longer term outcomes, you would want to know that the tumor characteristics at diagnosis were no worse, the interval cancer rate was no worse, the risk of lymphedema through aggressive node dissection was no worse, and the need for toxic therapies was no worse.

**Response:** We appreciate the thorough suggestions and comments from the reviewer. We accommodated the text in the manuscript to include his suggestions. Please, refer to text marked in red throughout the manuscript.

New Text (Introduction, page 5): “Overall, it is expected that sensitivity will be increased in the highest-risk group without significantly compromising specificity (false positives), and specificity will be increased in the lowest-risk group without significantly compromising sensitivity, particularly in the detection of early-stage breast cancers.”

Page 16 line 365: I have had a look at the MyPeBS website and have reviewed the background materials. It is rather remarkable that this study puts so much faith in risk estimation as offering a sweeping opportunity to improve outcomes, when the only thing that is reasonably certain is there are likely to be better outcomes from identifying a higher-risk group (including women at higher-risk that were not known to be), and screening them more aggressively. The opportunity to avoid the harms of screening in a lower-risk group focuses on reducing the opportunity for harms by reducing the exposure to screening, which includes the diagnosis of cancers that have a higher-risk of being overdiagnosed, for which there is zero evidence of that potential, or to be treated less aggressively, for which all women, lower and higher risk, might benefit if the cancer is localized and low grade.

**Response:** We appreciate this comment from the reviewer. We believe that this is slightly outside the scope of this qualitative study. However, we have modified the text in the manuscript to accommodate some discussion along this line of argument.

New Text (Discussion, page 16): “This study was conducted in the context of the MyPeBS study, which aims to improve the evidence on the effectiveness of a personalised screening strategy based on individual risk by increasing sensitivity in women at higher risk without compromising the diagnosis of early stage breast cancers in women at lower risk.”

Page 18, Line 411: The authors write, “Two European studies suggest that fear levels are high, even among women younger than 50 years, prompting them to undergo screening mammograms, even when not medically indicated and despite a high level of knowledge about the disease [22,23].” I think it would be less ideological to replace “even when not medically indicated,” with something that simply stresses in these countries screening before age 50 was not offered by the national service. There are numerous countries that offer screening to women in their 40s, and the evidence shows that screening is effective in this group. Saying it is not medically indicated implies that absolute risk is too low, or that screening is ineffective. Data from England and the US have shown clearly that there are only small differences in the burden of disease among women ages 45-49 and 50-54. Even if the country does not offer screening before age 50, it is not unreasonable for women to want to begin screening earlier. And, for some women, personalized screening would recommend that.

**Response:** As noted by the reviewer, we have modified the text to accommodate the reviewer’s suggestion.

New Text (Discussion, page 18): “Two European studies suggest that despite a high level of knowledge about the disease fear levels are high, prompting them to undergo more frequent screening, or even seeking for early detection when population based screening is not offered in women younger than 50 years.”

Page 20, line 451: Another essential and pressing issue raised in the study [is the training of primary care professionals [is the need to train primary care professionals], given the decisive role they will play in implementing a personalised screening model [30]. In this sentence, the need for training is stressed twice….I think the authors changed the way they wished to express this, and neglected to delete the text they wished to replace.

**Response**: We thank the reviewer for pointing out this mistake. We have modified the text accordingly.

**New text (Discussion, page 20)**: Another essential and pressing issue raised in the study is the need to train primary care professionals, given the decisive role they will play in implementing a personalised screening model [30].

General comment: One worrisome aspect of MyPeBS, and the narrative of this paper, is what feels like a high degree of confidence the personized screening will be shown to be effective for both higher and lower risk women, in effect that the interventions being tested will be shown to be efficacious for both groups. Although the authors state early on that noninferiority has not been demonstrated, and that this is the purpose of myPeBS, there seems to be little doubt about the outcome and the need to prepare for it. I agree that too often there is no preparation for the outcome of a large trial, and this delays implementation, but at this time, there should be some acknowledgement that the implementation challenges are uncertain overall, and that with respect to screening intervals, they may be shortened for higher-risk women, but not for lower-risk women. On line 434, the authors state, “Therefore, to promote acceptability among women deemed to be at low risk, several recommendations made by other researchers should be considered:…” Where there are statements like, this, I would encourage the authors to simply acknowledge, “if the findings from MyPeBS support wider screening intervals for lower-risk women, then……” Otherwise, it appears that moving ahead involves persuading women to accept a wider screening interval as a desirable trade off to reduce harms, in other words, taking a chance that safety will not be compromised in their life vs. the greater certainty of recalls (with the usually cited false positives, biopsies, and overdiagnosis) with more frequent screening, becomes the implementation challenge. Not only would that be debatable, but because the study is still underway, we’re not there yet, and it conveys that this would be the plan whatever the outcome.

If it becomes evident that lower-risk women can be screened safely at wider intervals, who among us wouldn’t want that? And, we (scientists and clinicians) would face a challenge persuading women that this is not about cost saving, but rather better outcomes. But, the final results of the study are not in. This study has identified what the investigators will face depending on the results. I encourage the authors to be clear that the study outlines the concerns of health professionals and what they perceive as their patients’ concerns, which will need to be taken into consideration depending on MyPeBS outcomes.

**Response:** As suggested by the reviewer, and in line with previous comments, we have modified the text in the manuscript accordi8ngly to accommodate the comments.

**New text (Discussion, page 19)**: If the findings from MyPeBS support wider screening intervals for lower-risk women, we need to promote acceptability among these women with the use of various tools and the adaptation of styles and formats to the levels of health literacy to provide information on personalisation and communicate risk [18,26].
